# Study on the Regulation Mechanism of Quality Deterioration Due to Chilling Stress and Dry Exposure during Anhydrous Storage and Transportation of Yesso Scallop *Patinopecten yessoensis*

**DOI:** 10.3390/foods12152902

**Published:** 2023-07-30

**Authors:** Peihong Jiang, Dongjie Chen, Xiangyang Chang, Changfeng Zhang, Xiuping Fan, Xiaoming Qin

**Affiliations:** 1 College of Food Science and Technology, Guangdong Ocean University, Guangdong Provincial Key Laboratory of Aquatic Products Processing and Safety, Guangdong Provincial Engineering Technology Research Center of Marine Food, Guangdong Province Engineering Laboratory for Marine Biological Products, Zhanjiang 524088, China; jiangpeihongjph@163.com (P.J.);; 2 Shandong Provincial Key Laboratory of Agricultural Products Storage, Transportation and Preservation Technology, Shandong Institute of Commerce and Technology, National Engineering Research Center for Agricultural Products Logistics, Shandong Guonong Logistics Technology Co., Ltd., Jinan 250103, China

**Keywords:** ecological ice temperature, chilling stress, anhydrous live preservation, regulation mechanism, *Patinopecten yessoensis*

## Abstract

In this paper, the quality change of Yesso scallop (*Patinopecten yessoensis*) in the process of anhydrous storage and transportation after cold acclimation and induced dormancy was studied, and the regulation mechanism of quality degradation during storage and transportation in the process of gradient chilling stress and drying exposure was further explored. The results show that, when transferred from hydrous to anhydrous states, the breathing pattern of the scallops changed from aerobic to anaerobic. Their gill filaments were altered and their apparent vitality constantly declined, which was reflected by the edge shrinkage of the pallium and the direct proportions of the edge reduction rate and the stimulus response period. After being in the anhydrous state for 4 d, the AEC value dropped to 67.59%. At this time, if they were placed under hydration again, the scallops resumed a good growth state. By proteomics analysis, it was revealed that cold acclimation and dry exposure mainly led to changes in biological functions and pathways, such as mitochondrial inner membrane and ATP hydrolysis activity. In addition, it can be seen from the functional annotation and enrichment analysis of the metabolite KEGG that cold acclimation promoted the purine metabolism of scallops, while dry exposure inhibited the metabolism of saturated fatty acids. In this study, the infrared sensing mode was used for the first time, too, in order to record the heart-rate changes of the scallops during circulation, which shows that non-destructive vitality monitoring of *Lamellibranchia* is feasible.

## 1. Introduction

Yesso scallop (*Patinopecten yessoensis*), as an important economic shellfish variety bred in northern China, has become the product of a leading industry that now drives the development of the local fishing economy [1,2]. Based on the dietary consumption habits of Chinese residents, vitality and freshness are the two main criteria to judge the quality of aquatic products, as well as the primary factors to consider when purchasing aquatic products. This has endowed fresh products with higher economic value due to their plump, tender, and delicious meat quality, as well as their plentiful and diversified nutrition [3]. Before going out into the market, scallops will go through four stages––that is, catching, clean-up and temporary breeding, anhydrous storage, and, finally, sale [4]. Therefore, issues such as optimizing and improving the transportation methods of aquatic products, enhancing the survival rate, reducing nutrient loss, and maintaining the original flavor quality are all urgent issues that need to be solved in industrial upgrading.

Ecological ice temperature anhydrous live preservation technology is a burgeoning method to drive aquatic animals into a dormant state at low temperatures, which can reduce their metabolic rate and thereby enable live preservation transportation to be conducted under low temperatures without the use of water [5,6]. Compared with traditional hydrous transportation, ecological ice temperature anhydrous live preservation manages to improve transportation efficiency and lower the cost of logistics, but the influence of stress factors, such as low temperature, cold acclimatization, hypoxic exposure, and desiccation, in waterless transportation on both the physiological state and the flavor quality of shellfish should be taken into consideration [7]. Many studies have shown that drastic changes in temperature can seriously affect the physiological activities, the immune function, and the disease resistance of shellfish, and can result in the death of the shellfish due to immune suppression [8]. Henzong et al. held the view that, before anhydrous live preservation, if Pacific oysters were trained in coldness at the ecological ice temperature, the effect of temperature stress on the oxidative immune system and the metabolism of energy substances could be reduced [9]. Lixin et al. simulated the vitality of scallops during circulation by analyzing the energy content, such as adenosine triphosphate, and they found that the vitality of scallops decreased for 24 h [10]. Hypoxia stress has a significant impact on the physiological metabolism and organizational structure of scallops. Indeed, Zhang et al. found that, after hypoxia treatment, scallops evinced decreased vitality, disturbed physiological and metabolic activities, and an abnormal organizational structure [11]. High temperature stress caused by increasing seawater temperature significantly affects energy metabolism, too [12]. However, there has been no systematic report on the stress regulation mechanism caused by stress, such as chilling stress and anhydrous storage. In this study, we examine the effects of environmental stress on the bodies of scallops, and we examine the effect of environmental stress in cold acclimatization and anhydrous storage and transportation in the temporary breeding stage.

## 2. Materials and Methods

### 2.1. Experimental Raw Materials

Scallops (*Patinopecten yessoensis*) cultured in net cages in Nanguang Island, Shandong, China, with a shell length of (76.30 ± 6.48) mm, a shell height of (18.97 ± 1.86) mm, and a wet weight of (59.63 ± 12.76) g, were selected as raw materials. In April 2022, immediately after being caught, they were sealed with foam boxes (ice pack cooling) and sent to Shandong Provincial Key Laboratory of Agricultural Products Storage, Transportation and Preservation Technology. Those that arrived in the laboratory in the living anhydrous hypoxia stress state were immediately placed in artificially circulated seawater for clean-up and were bred temporarily at a water temperature of (15 ± 2) °C and a salinity of 32‰. After temporary breeding, after 7 d, the scallops with strong vitality and an excellent state of health were selected to carry out the experiment.

### 2.2. Experimental Design and Methods

After temporary breeding in the laboratory, healthy and dynamic scallops were selected for gradient cooling and cold acclimatization to ecological ice temperature. In other words, by setting the cooling rate of the temporary breeding system to 2 °C/h, after lowering the temperature every 1 h, they were maintained at a constant temperature for 3 h. The temporary breeding temperature of 15 °C was lowered to a dormant temperature at 4 °C over 24 h and then the scallops were removed for anhydrous gas conditioning packaging. A dozen scallops were set in a group in a (335 × 220 × 175) mm foam box, with a biological ice bag placed at the bottom. Meanwhile, a temperature insulation bag was put between the ice bag and the scallops. The packed scallops were stored and transported in the low-temperature vibration chamber at 4 °C for 72 h, after which they were awakened in water in order to place the scallops after anhydrous storage and transportation into the temporary breeding system. Meanwhile, by the system program, the temperature was increased from the ecological ice temperature at a rate of 2 °C/h. After heating for 1 h, they were maintained at a constant temperature for 3 h until it returned to 15 °C, after which relevant index detection was performed.

### 2.3. Analysis of the Test

#### 2.3.1. Living Health Evaluation

Edge shrinkage: The edge shrinkage rate refers to the method of Li et al. [13], where edge shrinkage rate = edge shrinkage distance/shell height × 100%.

Stimulus response time: Pointed tweezers were used to stimulate the pallium of the live scallops and the contraction response time was recorded using a stopwatch.

Heart rate: The measurement method was slightly modified according to that of Bakhmet et al. [14]. First, the scallop shell was cleaned, an infrared sensor was attached to the outside of the scallop shell near the heart, and the scallop was placed in artificial seawater at 15 °C, which would then be detected after the antennas of the pallium were fully displayed. The Power Lab instrument parameter range (2~5 V) was set with a low pass (1~10 Hz) and AC coupling. LabChart8 was utilized to record the changes in scallop heartbeat stability and to intercept the area of the 2 auricular appendages and the 1 ventricular waveform, thus obtaining a complete and smooth waveform map. The stable waveform in 10 min of detection time was then calculated and 5 scallops were checked each time, totaling 3 times in parallel.

#### 2.3.2. H&E Staining and Histological Examination of Gill Tissues

The scallops were placed on a dissecting table to remove gill parts for H&E staining. All samples were cut into 5–6 µm thick slices. Then, H&E staining was performed for microscopic observation. Dehydration and transparency were performed in ethanol and xylene, and samples were then cover slipped. The stained sections were observed and photographed using a light microscope (Nikon, Tokyo, Japan, DS-Fi2) and spliced into a complete image using ImageJ software.

#### 2.3.3. Nucleotides and Associated Compounds

The scallop adductor muscle was dissected, extracted, and mashed by adding 5% PCA solution in an ice bath. The pH was adjusted to 2–3.5, 4000× *g* rpm for 5 min of centrifugation in order to obtain the supernatant, which was then filtered using a 0.45 μm membrane filter and analyzed by HPLC after adding phosphate buffer. The chromatographic conditions were column symmetry C18 (4.60 mm 150 mm, 5 μm); mobile phase A: 0.05 mol/L KH_2_PO_4_-K_2_HPO_4_ (pH 6.78); mobile phase B: chromatographic methanol; detection wavelength: 259 nm; sample intake: 20 μ L; flow rate: 1 mL/min; column temperature: 40 °C; and gradient elution. According to the peak time of the standard product, the composition of the sample was qualitatively determined, and the substance content was calculated from the peak area.

The nucleotide energy charge AEC (adenylate energy charge) value is an indicator reflecting the degree of environmental stress on animals, which can reflect the freshness of scallops. AEC(%) = (2ATP + ADP)/(2(ATP + ADP + AMP)) × 100%.

#### 2.3.4. Proteomics

The adductor muscle at different treatment stages was selected for proteomic analysis. Samples then underwent protein extraction, enzymatic peptide digestion, liquid chromatography–mass spectrometry (LC-MS) data collection, database retrieval, and bioinformatics analysis. Chromatography–mass spectrometry detection conditions were as follows––mobile phase A: formic acid aqueous solution; mobile phase B: 0.1% (*v*/*v*) formic acid solution. Peptide fragments were dissolved by liquid chromatography in mobile phase A and they were separated using an ultra-efficient liquid phase system. The liquid phase gradient setting was 0~120 min, 8~100%, and the mobile phase B flow rate was 300 nL/min.

#### 2.3.5. Metabolomics

The adductor muscle at different treatment stages was selected for metabolomics analysis. After shock crushing and cold sonication, the samples were centrifuged at 12,000× *g* rmp for 10 min at 4 °C, and the supernatant was removed for machine testing. The column was a C18 column (Zorbax Eclipse C18 (1.8 μm × 2.1 mm × 100 mm), and the chromatographic separation conditions were a column temperature of 30 °C and a flow rate of 0.3 mL/min, with mobile phase compositions A (0.1% formic acid solution) and B (pure acetonitrile, gradient elution).

In positive mode, the heater temperature was 325 °C; the sheath gas flow was 45 (arbitrary units); the aux. gas flow was 15 arb; the sweep gas flow was 1 arb.; the electrospray voltage was 3.5 KV; the capillary temperature was 330 °C; and the S-Lens RF level was 55%.

In negative mode, the heater temperature was 325 °C; the sheath gas flow was 45 arb; the aux. gas flow was 15 arb; the sweep gas flow was 1 arb.; the electrospray voltage was 3.5 KV; the capillary temperature was 330 °C; and the S-Lens RF level was 55%.

For the scanning mode, full scan was *m*/*z* 100~1500 and data-dependent mass spectrometry (dd-MS2, TopN = 10), and the resolution was 120,000 (MS1) and 60,000 (MS2). The collision mode was High Energy Collision Dissociation (HCD).

### 2.4. Data Processing

The experimental results are expressed as the mean ± standard deviation (X ± SD), the experimental data were processed using Origin2021 and SPSS27 software and analyzed by univariate analysis, and the significance level was set as *p* < 0.05.

## 3. Results

### 3.1. The Apparent Vitality of Patinopecten yessoensis Decreased during Anhydrous Storage and Transportation

**Figure 1:**
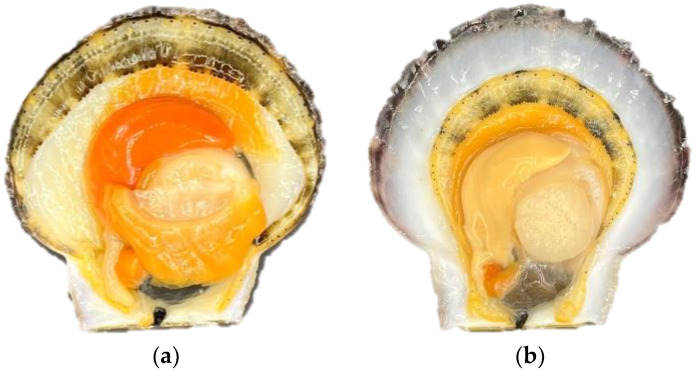
Living edge shrinkage of *Patinopecten yessoensis* in different periods: (**a**) temporary breeding and cold acclimatization; (**b**) anhydrous storage and transportation (3D).

**Figure 2:**
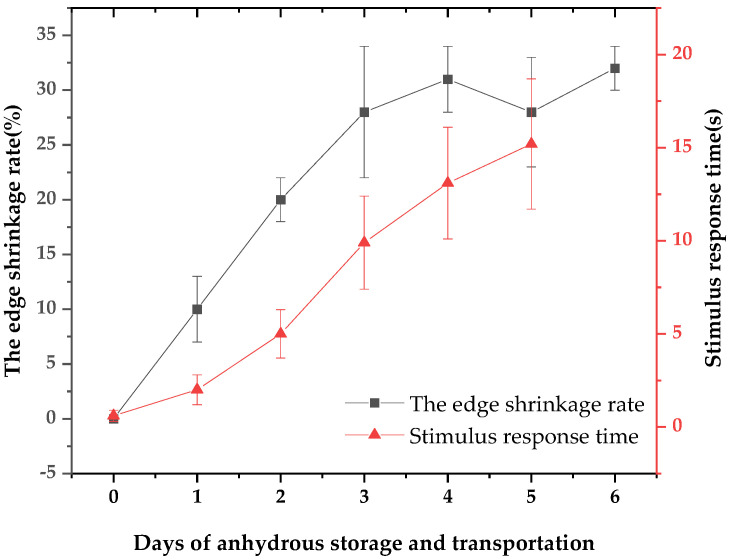
Changes in the apparent vitality index during anhydrous storage and transportation.

The apparent vitality of *Patinopecten yessoensis* was evaluated by determining the edge shrinkage rate and stimulus response time. During the period of purification and the temporary breeding and cold acclimatization, the pallium of the living scallops was full and had no shrinkage, and the tentacles fully extended to the shell. However, during the storage and transportation stage, as time went by, the edge shrinkage phenomenon appeared and the rate then gradually increased. By the third day of anhydrous storage and transportation, the rate reached 28.05% (Figure 1). Meanwhile, it was revealed by a Pearson correlation analysis of the pallium stimulus response time that the correlation coefficient was 0.914, showing a significant positive correlation (*p* = 0.011 < 0.05)––that is, the worse the contraction phenomenon, the worse the living state and the longer the stimulus response time (Figure 2).

### 3.2. Patinopecten yessoensis Vitality Dropped during the Anhydrous Storage and Transportation Process

**Figure 3:**
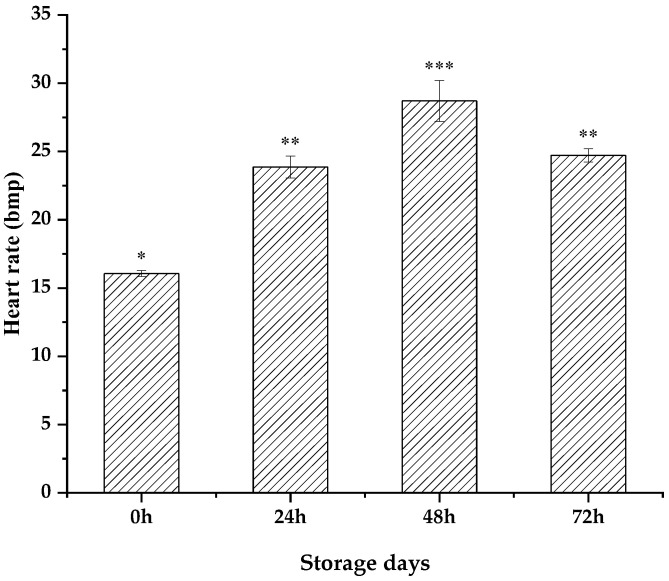
Heart-rate change of *Patinopecten yessoensis* after rehydration at different storage times. the significant difference was indicated by different symbos (*, ** and ***).

Infrared sensing was employed to monitor the heart-rate activity of scallops without doing any damage and the heart-rate cycle and intensity were recorded. The BMP value was the number of heart beats per minute. In our preliminary research, it was found that, during the 15 °C temporary breeding period, the average heart rate of scallops was 18.39 times/min. Gradient cooling was used to monitor the heart rate in real time. With the decrease in temperature, the heart rate gradually decreased and the heart rate dropped to 7.39 times/min at the cooling endpoint of 4 °C [15]. After 15 °C of rehydration, the heart rate was significantly higher than that of the control group (*p* < 0.05) (Figure 3). Among them, the heart-rate values at 24 h, 48 h, and 72 h were 23.86 ± 0.80 bpm, 28.70 ± 1.51 bpm, and 24.72 ± 0.48 bmp, respectively, which were 60.41% higher than those of the control group; the survival rate of scallops decreased after 72 h and the heart-rate measurement was thus terminated.

### 3.3. Changes in Microorganizational Structure of the Gills

**Figure 4:**
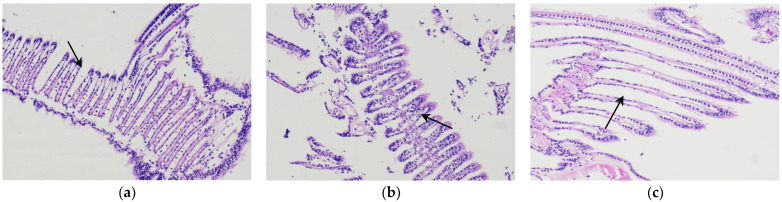
Microstructure of *Patinopecten yessoensis* gills at different stages (200), changes in gill filaments are marked in the figures: (**a**) temporary breeding stage; (**b**) cold acclimation stage; (**c**) no water storage for 3 d.

In the comparison of the microscopic tissue sections of the gills after temporary breeding, cold acclimatization, and water storage for 3 d (see Figure 4), it can be seen that the filaments of the gills in the clean-up and temporary breeding period were uniform and neat in arrangement. After gradient cooling cold acclimatization treatment, they became shorter and thicker. After being transferred from the water environment into the anhydrous storage environment, the scallops closed their shells to retain moisture, thereby reducing their oxygen intake. As was the case from oxygen breathing to oxygen respiration, low oxygen stress led the filaments to increase in space (i.e., extend) in order to increase the contact area for air exchange. Duan et al. reported that the respiration burst of Japanese *Penaeus orientalis* in an anhydrous environment for 3 h showed that respiration weakened at 10 h and, eventually, the gill cavity movement slowed down, leading to death [16].

### 3.4. Nucleotide-Lineage Compounds

**Figure 5:**
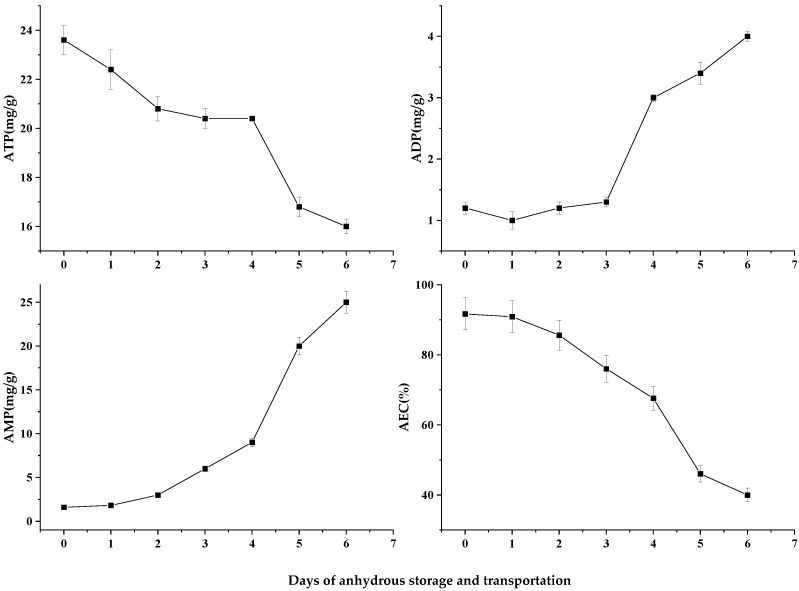
Changes in nucleotide series of *Patinopecten yessoensis* during anhydrous storage and transportation.

ATP provides the energy necessary to maintain normal life activities and the change in its content can better reflect the life status of shellfish [17]. In the process of anhydrous storage, the ATP content of *Patinopecten yessoensis* decreases by ladder (Figure 5). However, when the *Patinopecten yessoensis* changed from a hydrous to an anhydrous storage environment after cold acclimation, its breathing mode changed from aerobic to anaerobic and the release of energy was reduced, so the ATP showed a decreasing trend [18]. During 2–4 d of anhydrous storage and transportation, scallops were in a stress state for a long time, and they started their own stress response mechanism to mobilize energy materials and to maintain body balance, so the ATP content maintained a stable state in this stage. However, in the later period of anhydrous storage and transportation, with the gradual depletion of glycogen and other substances, the rapid ATP degradation showed a steep decline. Meanwhile, the decomposition of ATP led to an increase in ADP content. ADP is the substrate of the adenylate kinase reaction, producing one ATP and one AMP from two ADP. The changes in the ADP and AMP contents are shown in Figure 5. With the extension of anhydrous storage and transportation, the body could not maintain the relative stability of ATP content in an anoxic environment––that is to say, there was an occurrence of ADP and AMP accumulation.

Nucleotide-energy AEC is widely used in the vitality and quality evaluation of live fish and shellfish. Maguire et al. revealed that the AEC value in scallop muscle could effectively reflect the stress intensity and state of the scallop at that time, and that the AEC value was divided into three stages to show the vitality of shellfish in different periods: 80–90% indicates a good state and reproduction being available; 50–70% represents slow growth but recoverable reproduction; and less than 50% means irreversible damage [19,20]. At 4–5 d, the AEC values were 67.59% and 46.02% (Figure 5), respectively. Therefore, to ensure a good growth state of scallops after rehydration, the storage and transportation time should be controlled within 4 d.

### 3.5. Environmental Stress Induced Changes in the Proteome

In order to obtain the differential protein expression changes of scallops in three different stages––fresh temporary breeding storage (XHZ), cold acclimation (LXHZ), and anhydrous live preservation storage (BHZ)––the non-labeled quantitative proteomics technique was used in this study. A search of the UniProt database identified 4548 peptides, 4134 unique peptides, and 856 proteins (Table 1). Based on the above data, a systematic bioinformatics analysis of quantitatively informative proteins was carried out, including protein annotation, significant difference analysis, annotation clustering based on significant difference, and protein interaction network analysis, in order to provide reference directions for in-depth study of the proteome.

#### 3.5.1. GO Annotation

**Figure 6:**
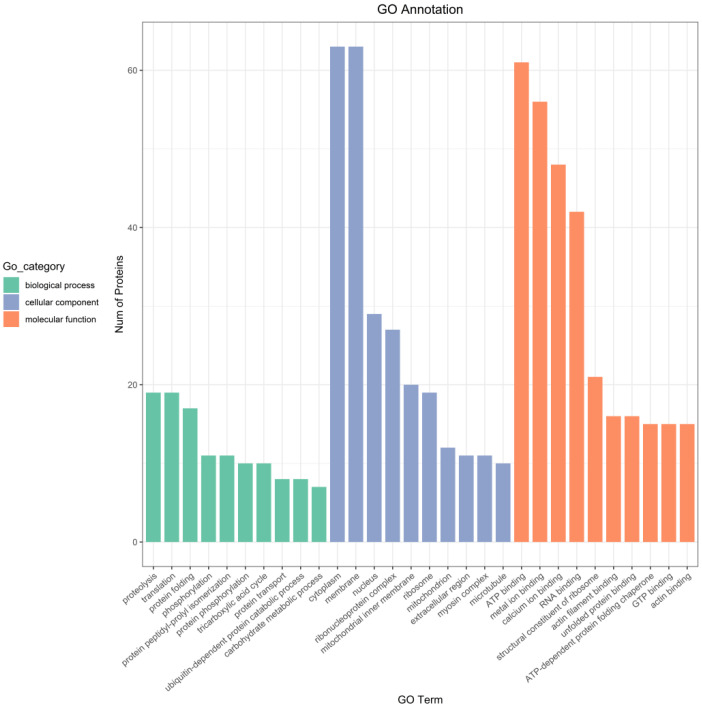
Bar graph of the GO annotation results.

GO (Gene Ontology) refers to an internationally standardized classification system for gene function description, which is divided into three categories––namely, the Cellular Component, which is used to describe subcellular structure, location, and macromolecular complexes; the Molecular Function, which is used to describe the function of individual gene products; and the Biological Process, which is used to describe the biological process in which the gene-encoded products participate. According to the GO annotation results, the number of proteins corresponding to different GO entries was counted, and the annotation results of the top 10 in each major class of the GO database were drawn. As seen in Figure 6, their biological functions were mainly focused on proteolysis, translation, protein folding, phosphorylation, and other aspects, which are mainly involved in cytoplasm in terms of cellular components, membrane, ribosome, mitochondrion, and other structures. In terms of molecular functions, they were ATP binding, metal ion binding, structural constituents of ribosome, etc.

#### 3.5.2. COG Annotation

COG (Cluster of Orthologous Groups of proteins) refers to the protein database created and maintained by NCBI, which was constructed based on the phylogenetic relationship classification of the coding proteins of the complete genomes of bacteria, algae, and eukaryotes. Through the alignment, a certain protein sequence can be annotated to a certain COG and each cluster of COG is composed of a direct homologous sequence, so that one can speculate on the function of the sequence. The COG database is divided into 26 categories according to its functions and its annotation bar charts are drawn according to the annotation results, with the results showing that the identified proteins are mainly concentrated in post-translational modification, protein turnover, and chaperones, as well as signal transduction mechanisms, cytoskeletons, energy production and conversion, and other processes.

#### 3.5.3. Analysis of Variance

**Figure 7:**
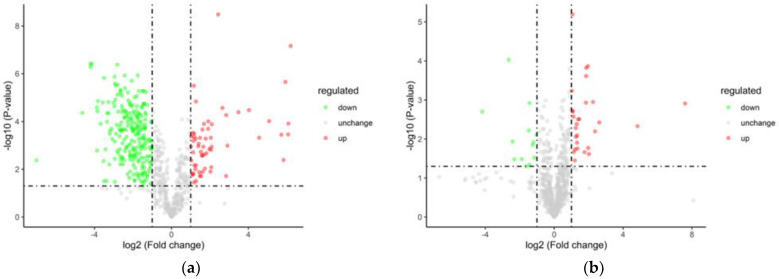
Volcano plots of the differential proteins: (**a**) LXHZ vs. XHZ; (**b**) BHZ vs. LXHZ.

Fresh temporary breeding (XHZ), cold acclimation (LXHZ), and anhydrous live preservation storage and transportation (BHZ) were used for between-group comparison, in which the ratio of all of the biological replicates in the comparison samples was taken as the multiple of difference (Fold Change, FC) and the protein was increased when FC ≥ 2 (logFC ≥ 1), while, when FC ≤ 0.5 (logFC ≤ −1), the protein showed downregulation of expression. Among them, the cold acclimation group upregulated 60 and 237 differentially expressed proteins compared with the transient group. It was speculated that scallops inhibited the enzyme activity by cold acclimation, so the number of downregulated expressed proteins was higher. However, the unwatered storage group raised 28 differential proteins and lowered 13 differential proteins compared with the cold acclimation group (Table 2), and Figure 7 shows volcano plots of the differential proteins.

#### 3.5.4. GO Annotation and Enrichment Analysis of Significantly Differential Proteins

**Figure 8:**
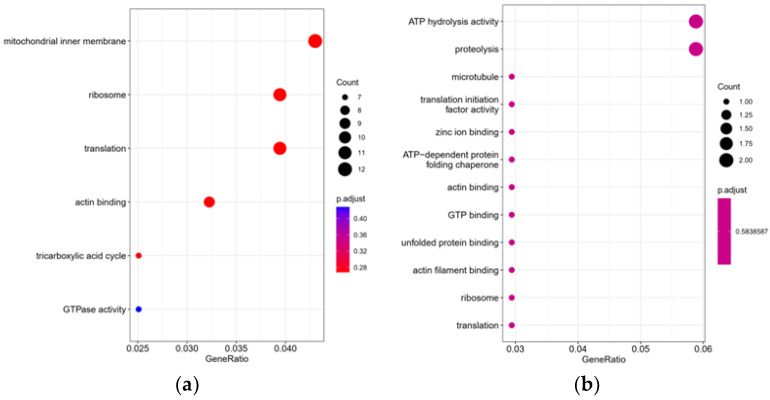
GO enriched bubble diagram of differential proteins: (**a**) LXHZ vs. XHZ; (**b**) BHZ vs. LXHZ.

GO function significance enrichment analysis identified the GO function entries significantly enriched in the differential proteins compared to all of the identified protein backgrounds, thus providing us with the biological functions that the differential proteins are significantly associated with (*p* < 0.05). Based on the enrichment results, the bubbles of the enriched GO entries were drawn (Figure 8). The results showed that, after gradient cooling, the differential proteins were mainly enriched in mitochondrial inner membrane, ribosome, translation, actin binding, and other biological functions and pathways, while, in the transition from cold acclimation to the no water storage stage, the body mainly showed significant changes in ATP hydrolysis activity and proteolysis.

### 3.6. Environmental Stress Induced Changes in the Metabolome

**Figure 9:**
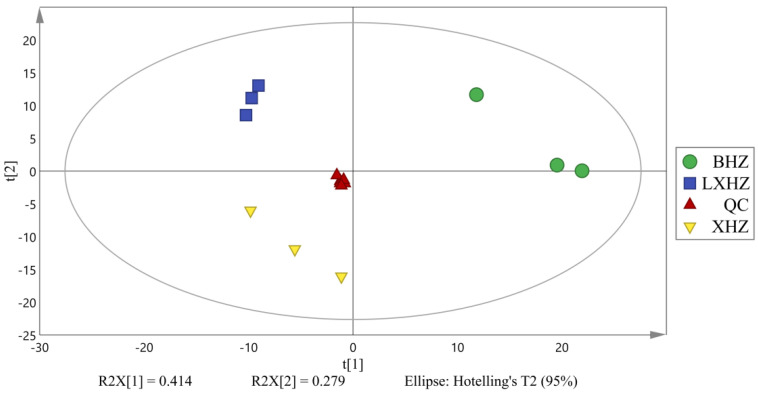
PCA diagram of mass spectrometry data of three scallop samples and mixed samples (XHZ—temporary breeding group, LXHZ—cold acclimation group, BHZ—anhydrous storage and transportation group, and QC—quality control mixed samples).

The complex metabolic reactions and their regulation in organisms are not conducted alone, and they often form complex pathways and networks involving different genes and proteins whose mutual influence and mutual regulation eventually lead to systematic changes in the metabolome. Global metabolites in and out of the cell were analyzed qualitatively or semi-quantitatively by non-target metabolomics in order to explore the effects of cold acclimation stress and anhydrous storage on the metabolic processes of scallops. Before conducting the difference analysis, principal component analysis (PCA) of the grouped samples for the difference comparison was undertaken in order to observe the variation size between the different groups and between the samples within the group. Figure 9 shows that the three experimental samples were very significant and could be used for subsequent analysis; almost all were within the 95% confidence interval, which indicates cold acclimation (unless storage and transportation had a significant impact on scallop metabolism).

#### 3.6.1. Differential Metabolite Screening

**Figure 10:**
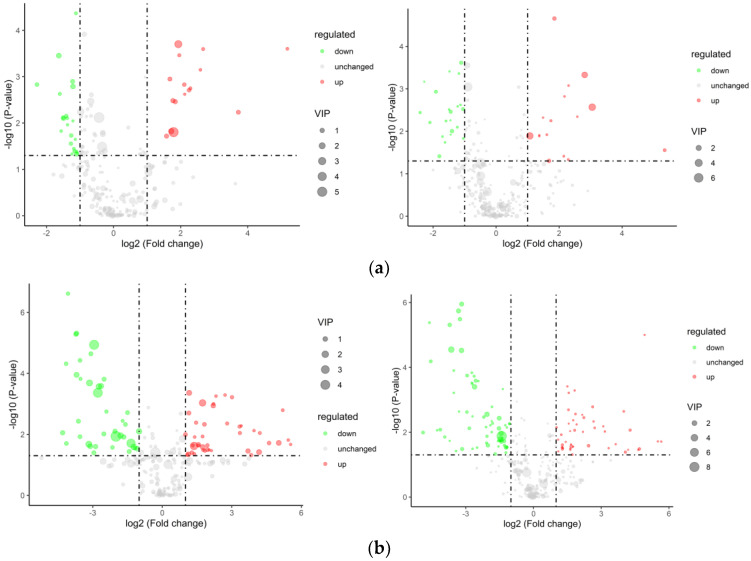
Volcano plots of the differential metabolite in positive and negative ion models: (**a**) LXHZ vs. XHZ; (**b**) BHZ vs. LXHZ.

Figure 10 shows volcano plots of the differential metabolites in positive and negative ion models. Comparing the differential metabolites between the transient breeding group and the cold acclimation group, 68 differential metabolites were obtained, including adenylosuccinic acid, adenylosuccinic acid, guanosine, and dodecyl sulfate, among which 30 were upregulated products and 38 were downregulated. The cold acclimation group was stored and transported without water for 3 d, which produced 179 differential metabolites, including DL-Arginine, myristic acid, adenylosuccinic acid, Tetradecanoyl-L-Carnitine, Oleoyl-L-Carnitine, and palmitic acid, of which 85 were upregulated products and 94 were downregulated.

#### 3.6.2. Functional Annotation and Enrichment Analysis of the Differential Metabolite KEGG

**Figure 11:**
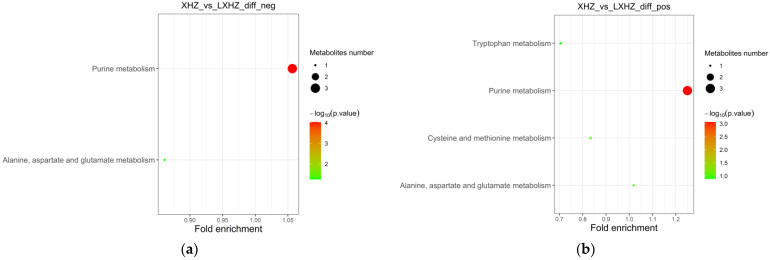
KEGG enrichment pathway of cold-acclimated differential metabolites: (**a**) negative ion mass spectrum; (**b**) positive ion mass spectrum.

**Figure 12:**
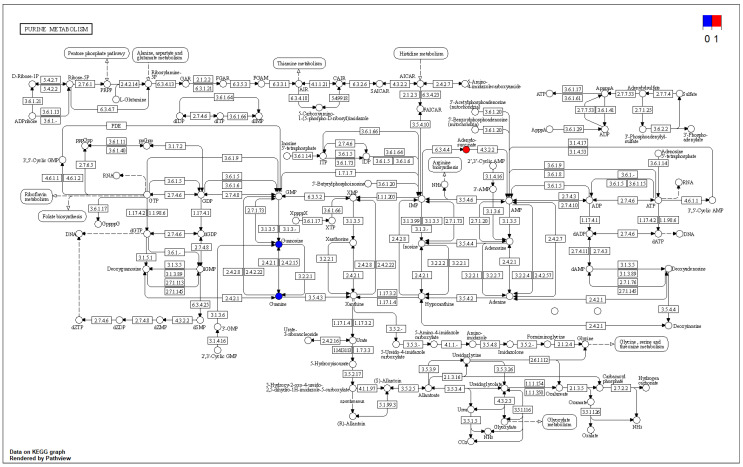
Metabolic pathway map of purine metabolism.

The differential metabolites were annotated through the KEGG database and classified based on the corresponding pathway; the size of the scatter in the figure indicates the number of differential metabolites that were enriched on the pathway. As can be seen in Figure 11 and Figure 12, the differential metabolites produced by the transient group by cold acclimation treatment mainly belonged to the purine metabolism pathway of KEGG. Cold acclimation promoted the purine metabolism of the scallops, and the differential metabolites were labeled on the pathway map showing the content of adenylosuccinate and IMP, which were 3.60 times and 3.37 times that of the transient control, respectively, while the content of guanosine decreased significantly.

**Figure 13:**
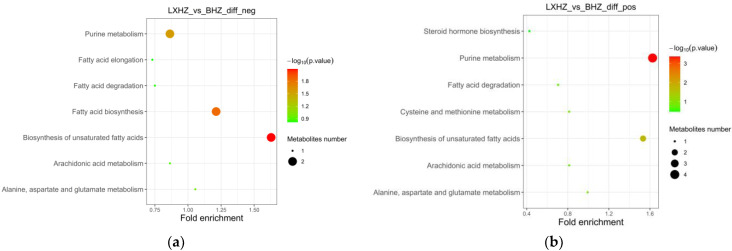
KEGG enrichment pathway of anhydrous storage and transportation differential metabolites: (**a**) negative ion mass spectrum; (**b**) positive ion mass spectrum.

The cold acclimation group was stored and transported without water for 3 d, and the differential metabolites generated at this stage mainly belonged to the biosynthesis of unsaturated fatty acids, fatty acid biosynthesis, and purine metabolism pathways of KEGG (Figure 13). It was then speculated that low-temperature and anhydrous storage and transportation inhibited the metabolism of saturated and unsaturated fatty acids. After making the differential metabolites visible in the pathway map, tetradecanoic acid, hexadecanoic acid, icosapentaenoic acid (EPA), and palmitic acid were significantly reduced.

## 4. Discussion

The flavor quality of scallops is closely related to their physiological state. In the living market, the opening rate, closure sensitivity, and mantle shrinkage often reflect the quality of the scallop [21]. Biochemical indicators, such as pH, glycogen, ATP-related substances, and K values, are also widely used in shellfish activity evaluation methods [22,23]. The apparent vitality of *Patinopecten yessoensis* evaluated by the rate of edge shrinkage and stimulus response time was consistent with the results of Li et al. The degree of the pallium was positively correlated with the stimulus response time. The greater the degree of edge shrinkage, the longer the stimulus response time––that is, the worse the vitality state.

When stressed by an adverse external environment, living animals can produce stress and cause changes in heart rate, and non-implantable heart-rate measurement is more and more widely used in marine organisms. Mat et al. detected the rhythmic activity of Pacific oysters by means of adhesion sensing, and Dong et al. studied the temperature and the heat resistance of limpets at different temperatures through heart-rate assessment methods. Chen et al. also used the method of heart-rate assessment to evaluate the temperature and the heat resistance of abalone, which shows that the method of heart-rate assessment is becoming increasingly mature in the study of shellfish.

It is well recognized that scallops usually upregulate the expression of a large number of stress proteins in order to cope with protein damage from environmental stress and this process obviously requires the expenditure of large amounts of energy [24,25,26,27,28,29]. Energy material is often the most direct reaction to the physiological state of the scallop. ATP gradually decreased in the process of anhydrous storage and transportation of *Patinopecten yessoensis*, and this was consistent with the earlier results of Zhang et al. [30,31]. Scallops are often in a state of hypoxia during long-term transportation and severe hypoxia will have a negative impact on the general health of the scallop. The loss of ATP under hypoxia stress also leads to a decrease in blood cell adsorption capacity and phagocytosis activity [32]. In Xu’s study, seven different metabolites were produced after wet storage and transportation and the pathway analysis showed that the tricarboxylic acid cycle was the most susceptible pathway [33]. Energy metabolism involves the degradation and synthesis of high-energy phosphates [34], and both GMP and IMP in this study might be related to this process. The results of proteomic GO annotation also showed that the differential proteins were mainly characterized in the tricarboxylic acid cycle, mitochondrion, and ATP, and this was consistent with the metabolome.

## Figures and Tables

**Table 1:** Protein quantitative results.

Database	Peptides	Unique Peptides	Protein Groups
*Patinopecten yessoensis* (Mizuhopecten yessoensis_6573)	4548	4134	856

**Table 2:** Differential protein quantity statistics.

Comparisons	Up	Down	All
XHZ_vs_LXHZ	60	237	297
LXHZ_vs_BHZ	28	13	41

## Data Availability

The data used to support the findings of this study can be made available by the corresponding author upon request.
